# The power of electrified nanoconfinement for energising, controlling and observing long enzyme cascades

**DOI:** 10.1038/s41467-020-20403-w

**Published:** 2021-01-12

**Authors:** Giorgio Morello, Clare F. Megarity, Fraser A. Armstrong

**Affiliations:** Inorganic Chemistry Laboratory, Department of Chemistry, University of Oxford, Oxford, OX13QR UK

**Keywords:** Enzymes, Biocatalysis, Electrochemistry

## Abstract

Multistep enzyme-catalyzed cascade reactions are highly efficient in nature due to the confinement and concentration of the enzymes within nanocompartments. In this way, rates are exceptionally high, and loss of intermediates minimised. Similarly, extended enzyme cascades trapped and crowded within the nanoconfined environment of a porous conducting metal oxide electrode material form the basis of a powerful way to study and exploit myriad complex biocatalytic reactions and pathways. One of the confined enzymes, ferredoxin-NADP^+^ reductase, serves as a transducer, rapidly and reversibly recycling nicotinamide cofactors electrochemically for immediate delivery to the next enzyme along the chain, thereby making it possible to energize, control and observe extended cascade reactions driven in either direction depending on the electrode potential that is applied. Here we show as proof of concept the synthesis of aspartic acid from pyruvic acid or its reverse oxidative decarboxylation/deamination, involving five nanoconfined enzymes.

## Abstract (web summary)

Multistep enzymatic reactions (cascades) can be achieved by confining enzymes in synthetic materials, but ways to simultaneously energize, control and observe the reactions in real time are lacking. Here, bidirectional interconversion between aspartate and pyruvate by a five enzyme cascade trapped in electrode nanopores, addressable by laptop commands, is demonstrated.

## Introduction

**Fig. 1:**
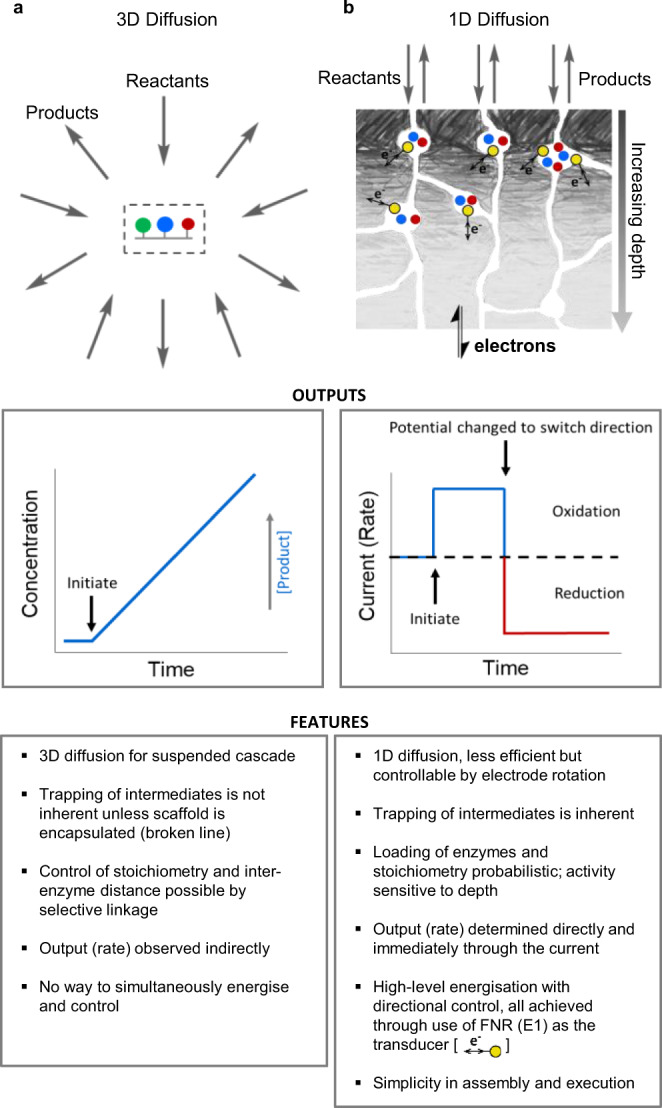
Comparing nanoconfined enzyme cascades. Comparisons between: **a** catalysis by a nanoconfined enzyme cascade immobilized on a scaffold that is suspended in solution; **b** catalysis by an enzyme cascade confined in zones of nanopores formed naturally by electrodeposition of indium tin oxide nanoparticles on a conducting support. The nanopore-confined cascades are energizable and directly observable via electron flow through the transducer ferredoxin-NADP^+^ reductase (E1).

In living organisms, the efficiency of multistep reactions (cascades) is optimized by confining enzymes within nanocompartments^1–5^. In such a way, the local concentration of each enzyme is massively increased, and in what is often termed substrate channeling, intermediates can be prevented from escaping and the distances they must travel between catalyst partners are minimized. Nanoconfining a package of enzymes in synthetic materials^6–20^ brings these advantages to new technologies where cascade reactions are already attractive^21–29^: furthermore, approaches such as directed evolution are producing individual enzymes for completely new functions to increase chemical complexity^30–32^. Enzyme cascades are valuable also for synthesis because they offer several advantages such as a decrease in purification steps and quick removal of inhibitory/toxic intermediates^25^. What is currently lacking (and would be invaluable) is a way of simultaneously energizing, controlling, and observing nanoconfined cascades in real-time, and we now describe how this can be achieved in a way that can greatly facilitate systems enzymology. To assist the explanations and discussions that will follow, our approach is compared in Fig. 1 alongside a consensus representation of current strategies for studying enzyme cascades arranged and confined on a nano- or micro-sized scaffold that is usually suspended in solution.

**Fig. 2:**
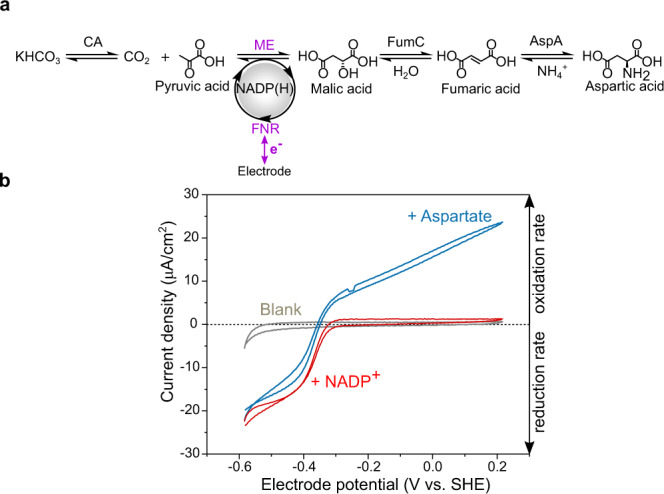
Cyclic voltammetry of an extended enzyme cascade trapped in electrode nanopores. **a** Scheme of the nanoconfined cascade driving reductive amination/carboxylation of pyruvate to aspartate in either direction through bidirectional electrocatalytic recycling of NADP(H) by the transducer enzyme FNR. CA carbonic anhydrase; FNR ferredoxin-NADP^+^-reductase; ME malic enzyme; FumC fumarase; AspA aspartate-amino-lyase. **b** Cyclic voltammetry (25 °C, pH 7.5, 1 mV s^−1^) of the 5-enzyme cascade (0.1 CA/1 FNR/5 ME/1 FumC/1 AspA) in buffer 0.05 M HEPES, 20 mM pyruvate, 0.1 M KHCO_3_, 0.1 M NH_4_Cl, 4 mM MgCl_2_, 1 mM MnCl_2_. Gray: blank, no cofactor present. Red: injection of NADP^+^ (to 20 µM). Blue: injection of aspartate (to 20 mM).

In this work, we demonstrate a cascade executed by five different enzymes confined and crowded within the nanopores and cavities of a thin film nanoporous electronically conducting oxide material (Fig. 1b), allowing complex biocatalysis to be managed at a simple electrochemical workstation. The cascade shown in Fig. 2a connects l-aspartic acid to pyruvic acid, ammonium ion, and bicarbonate. A linear sequence of four enzymes, E1 = ferredoxin NADP^+^ reductase (FNR, EC 1.18.1.2), E2 = l-malate NADP^+^ oxidoreductase (malic enzyme ME, EC 1.1.1 40), E3 = fumarase (FumC, EC 4.2.1.2) and E4 = l-aspartate ammonia-lyase (AspA, EC 4.3.1.1), is serviced by E2A = carbonic anhydrase (CA, EC 4.2.1.1) at a branching point next to E2. Carbonic anhydrase allows carboxylation from a bicarbonate salt, avoiding the need for gaseous CO_2_. The cascade is energized by direct electron transfer between the electrode and the FAD group of E1 (represented as yellow circles in Fig. 1b), which drives rapid and reversible interconversion of NADP^+^ and NADPH and acts here as the tranducer^33–38^.

## Results and discussion

### Bidirectionality of extended cascade observed by cyclic voltammetry

A first demonstration that long and complex biocatalytic cascades can be assembled, driven in either direction, and visualized in such a simple way, is given in Fig. 2b. The cyclic voltammograms—revealing how the steady-state catalytic velocity of the cascade varies with driving force—were obtained for a mixture of pyruvate, KHCO_3_, NH_4_Cl, NADP^+^, in the absence and presence of aspartate. They were measured after loading all the enzymes into a nanoporous layer of indium tin oxide (ITO) formed by depositing commercial nanoparticles electrophoretically onto a titanium foil support (area 1.89 cm^2^). The ITO layer, varying between 1 and 3 µm deep depending on the deposition time (2–10 min), comprises pores and cavities of less than 100 nm in diameter^33,35,36^. This procedure provides a fast, natural way of creating random nanospace (i.e., of ≪1 μm porosity) that can be energized electrochemically via the trapped FNR (E1), which serves as the transducer (Fig. 1b). The catalytic activity of a two-enzyme cascade was already established to depend upon the ability of each enzyme to enter and bind in these spaces^35^. The package was loaded as a 20 µL premixed solution having a ratio 0.1/1/5/1/1 (CA/FNR/ME/FumC/AspA), where we define 1 enzyme equivalent as 2 nmol. The solution was allowed to soak in for 20 min at room temperature after which the electrode was rinsed thoroughly with ultrapure water before placing it in the electrochemical cell (4 mL). A quantity of ~17 nmol total enzyme cm^−2^ was applied to the electrode, although only the uptake of FNR could be quantified: in this experiment, integration of its sharp, two-electron FAD redox signals (Supplementary Fig. S1) gave a value of 64.5 pmol cm^−2^, equating to a concentration of 0.65 mM FNR for a 1 µm layer, ignoring the volume taken up by the material^33,37^. The concentration established for FNR alone implies that, collectively, the nanoconfined enzymes can have a total concentration greatly exceeding millimolar and will thus be crowded together.

The gray trace shows the results before introducing NADP^+^ and aspartate into the surrounding solution (4 mL) of the electrochemical cell. The pH of 7.5 was chosen as a compromise with respect to reported pH optima for all the enzymes involved. Injection of NADP^+^ (to a final concentration of 20 µM) produced a large reduction current, then the addition of aspartate led to a bidirectional catalytic voltammogram. The sharp crossover point (at which there is zero net current) ideally equates to the reduction potential of the overall reaction for the entire cascade (Fig. 2b) and demonstrates the possibility for measuring, directly, the thermodynamics of elaborate and even de novo metabolic pathways, although this ideal situation depends on minimizing the escape of intermediates (see below). Similar results were obtained for an unbuffered solution (Supplementary Fig. S2) and for a four-enzyme cascade (with no CA) in the presence of 100% CO_2_ (Supplementary Fig. S3), although high CO_2_ levels are inhibitory in the oxidation direction. High CO_2_ levels also lower the cell pH unless heavily buffered: its localized production by CA in the nanopores avoids this problem.

### Upstream/downstream catalysis: hydrodynamic and potential control

**Fig. 3:**
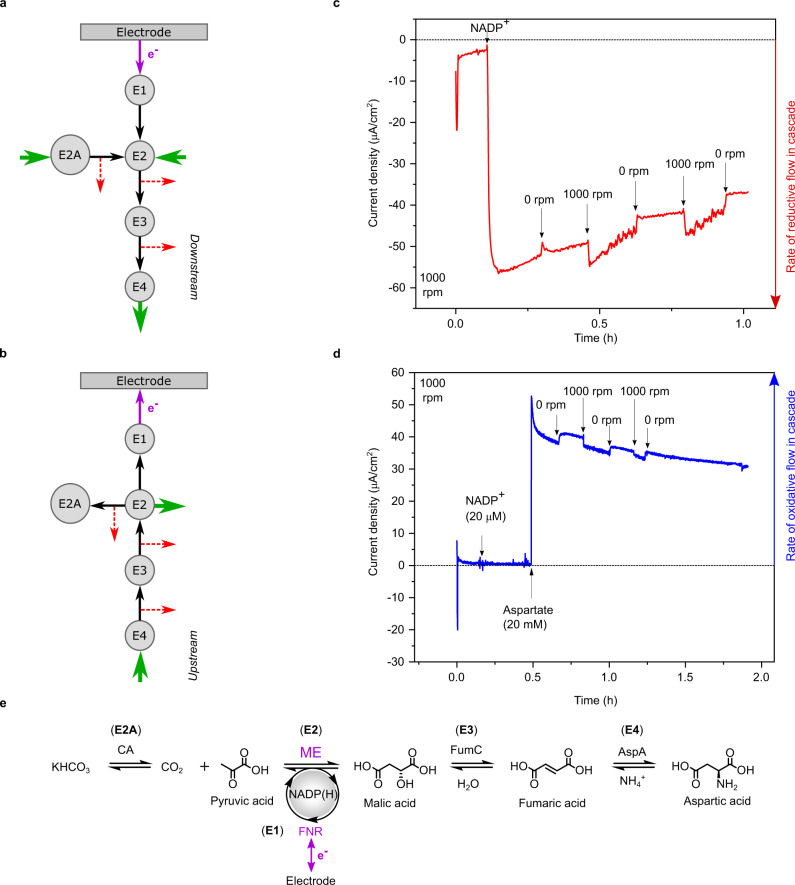
Controlling and monitoring the rate and directionality of an enzyme cascade. **a** The nanoconfined cascade in downstream notation. Enzymes E1 and E2 are, respectively, FNR and a NAD(P)(H) dehydrogenase and they represent the transducer-engine pair at which the rate of reaction flow along the cascade is recorded directly as current. Here the rest of the cascade is downstream with respect to the cofactor-dependent step (E1–E2 pair). Intermediates are passed to the next enzyme or escape (dotted red lines) into the bulk solution. **b** The nanoconfined cascade in upstream notation: the rest of the cascade is upstream of the cofactor-dependent step. **c** Rotation-rate dependence experiment for the system (CA/FNR/ME/FumC/AspA)@ITO/PGE (0.03 cm^2^) in a buffer containing NH_4_Cl, KHCO_3_, and pyruvate at pH 7.5, 25 °C, rotation rate 1000 rpm, under N_2_ atmosphere. Arrows signify injection of NADP^+^ (to a final concentration of 20 µM) and switching rotation off (0 rpm) and on (1000 rpm). **d** Continuation of the experiment carried out in panel c: the cell was washed carefully, a fresh buffer was added and rotation (1000 rpm) was switched on. Arrows signify injection of NADP^+^ (to 20 µM) and aspartate (substrate of E4, to 20 mM) and successive switching off and on of rotation. **e** Cascade with E1, E2, etc, identified with the different enzymes making up the cascade unit: downstream direction runs left to right.

The cascade can be driven in either direction simply by changing the electrode potential, which can be controlled by a simple command on a laptop computer (Fig. 1b: outputs). The terms Upstream and Downstream are introduced to describe where the cascade activity lies relative to the electron transfer that is occurring and being reported at the transducer FNR (E1): more widely, this notation is independent of whether an oxidation or reduction reaction is being driven. The E1–E2 pair converts the chemical flow rate through the cascade into electrical current and, like a river, events upstream of the E1–E2 pair are detectable whereas events downstream are not, unless inhibitory products build up. Figure 3a, b explains the concept: block green arrows indicate supply of reactants and release of desired product, black arrows represent unidirectional chemical flow internally through the cascade, and red arrows denote the escape of different intermediates to the cell solution, which competes with the catalytic flow through the cascade.

Figure 3c, d shows results of chronoamperometric experiments conducted with a rotating disk electrode in which the ITO layer has been deposited on a 0.03 cm^2^ graphite disk. In both cases, rapid electrode rotation increases the diffusion gradients at the electrode surface, thereby accelerating the supply of reactants or escape of intermediates. Figure 3c shows results for the downstream reaction (see Fig. 3e for enzyme and reaction-step identification), the synthesis of aspartate, which is driven simply by applying an electrode potential of −0.45 V vs SHE. Against a general slow decrease, the rate, observed as current, increases sharply (by up to 10%) each time the rotator is switched on, returning to the trend line when the rotator is switched off. This behavior is expected for a fast reaction because rotation increases the rate of supply of reactant molecules from the bulk solution. Figure 3d shows results for the upstream reaction, oxidation of aspartate. The experiment was conducted using the same electrode as used for panel c after changing the cell solution and setting the electrode potential to +0.1 V vs SHE. In contrast to the downstream reaction, the rate increases sharply (by ~5%) each time the rotation is stopped: the main effect of rotation is now to facilitate the escape of intermediates which decreases the current that is detected through the E1–E2 pair. Notably, the nanoconfined enzymes are not dislodged by the centrifugal force set up at the rapidly spinning electrode.

### Analysis of products released during catalysis in either direction

**Fig. 4:**
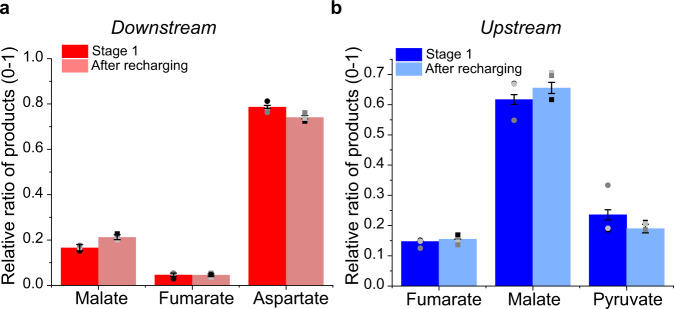
Analysis of products and intermediates. Relative percentage of products in the downstream direction (reductive amination/carboxylation) (**a**), and upstream direction (oxidative deamination/decarboxylation) (**b**). In both cases, the relative product distribution was maintained after recharging the system with new buffer and substrates. Error bars represent standard errors of the mean taken from three independent repeat experiments (data points plotted as circles for stage 1 and squares after recharging, *N* = 3).

We next addressed the overall performance through ^1^H-NMR analysis of products and intermediates over long time periods and under different conditions. Figure 4a shows the results for the synthesis of aspartate from pyruvate (20 mM), KHCO_3_ and NH_4_Cl carried out using an ITO/Ti foil electrode loaded with the cascade enzymes. The cell volume was 4.5 mL and the electrode area was 12 cm^2^: the enzymes were loaded in a different ratio 0.1/1/5/2/1 (CA/FNR/ME/FumC/AspA) as we expected this would increase the rate of flow along the cascade towards the product, aspartate. The electrode potential was held at −0.45 V vs SHE for a 20 h period (under continuous stirring); the solution was then removed, the cell recharged with reactants and 20 µM NADP^+^, and the reaction was continued for a further 20 h. The two current-time traces were in close correspondence, showing that the nanoconfined enzymes are stable over a useful time period and the decrease in rate with time is due to the accumulation of products (Supplementary Fig. S4). For the experiment shown in Supplementary Fig. S4 a conversion of 34%/24% pyruvate to aspartate was obtained for the first/second 20 h runs (Table 1, Expt 1), with the following product distributions: 6.80/4.8 mM aspartic acid, 0.06/0.02 mM fumarate and 1.4/1.26 mM malate (Supplementary Fig. S5 for NMR). Aspartate is the dominant product and there is relatively little release of malate and (even less so) fumarate intermediates. The total turnover numbers (TTNs) of aspartate/NADP^+^ formed were 340 and 240 for the first and second 20 h, respectively. Decreasing the NADP^+^ concentration to 5 µM produced a similar conversion: for example, in a 40 h experiment, 23.4% of pyruvate was converted to aspartate (Table 1, Expt 2) the TTN based on aspartate production being 936 (Supplementary Fig. S6). Clearly, NADP^+^ needs to be present only in catalytic amounts, although it exchanges between the pores and bulk solution. Figure 4b shows the analogous results for the oxidation of aspartate (Supplementary Fig. S7), where it is now clear that malate, not pyruvate, is the dominant product. The corresponding conversion figures achieved in that experiment—where the initial concentration of aspartate was 20 mM (in 4 mL)—were as follows: total (aspartate to pyruvate, fumarate, malate) 30% ± 4%; aspartate to pyruvate, 7.8% ± 0.9%; aspartate to malate: 18% ± 3.5%; aspartate to fumarate: 4% ± 0.7%.

**Table 1:** Results of downstream experiments for aspartate synthesis starting from pyruvate (20 mM) using 20 and 5 µM NADP^+^.

Expt	Substrates (mmol)		Products (mmol)			Parameters			
	Pyruvate	NADP^+^	Malate	Fumarate	Aspartate	Time	TTN	Conversion	From coulometry
1	0.09	9 × 10^−5^	0.0065	0.0003	0.0306	First 20-h	340	34%	0.036
	0.09	9 × 10^−5^	0.006	0.0001	0.0216	Second 20 h	240	24%	0.039
2	0.09	2.25 × 10^−5^	0.0035	0.001	0.0211	40 h	936	23.4%	0.029

Current traces are shown in Supplementary Fig. S4 (experiment 1) and Supplementary Fig. S6 (experiment 2).  Volume in all cases = 4.5 mL.

Enzyme concentrations applied to the ITO layer were varied over a factor of 50. The general product distributions seen in Fig. 4a, b persisted despite varying the amount of each enzyme applied to the ITO surface. Notably, FumC and AspA were required in lower amounts than the other enzymes (Supplementary Figs. S8 and S9). Experiments in which enzymes were deposited separately in different areas of the electrode showed no activity, confirming that the enzymes function as a cascade only when nanoconfined together in the pores (Supplementary Fig. S10). The presence of CA was essential for the downstream aspartate synthesis starting from bicarbonate but made no difference in the upstream oxidation direction (Supplementary Fig. S11). Aspartate is not electroactive at the potential applied (Supplementary Fig. S12).

To help interpret these results we measured the steady-state solution kinetics of E2, E3, and E4 under similar conditions to the electrochemical experiments: *k*_cat_^app^, *K*_M_^app^, and resultant specificity constants (*k*_cat_^app^/*K*_M_^app^) are shown in Table 2. Carbonic anhydrase is an extremely active enzyme, but its role lies only in the service branch E2–E2A. The local concentrations of each enzyme in the nanoporous layer probably exceed the local concentrations of intermediates, but *K*_M_^app^ is still a reasonable gauge of how well an enzyme will capture its reactant. From these data, the rate-determining step in downstream aspartate synthesis is catalyzed by ME, the least active of the enzymes. The high activities of fumarase (100×) and AspA (1000×) then ensure that most malate is taken forward catalytically and very little fumarate escapes, explaining why aspartate is produced in such a high yield. For upstream aspartate oxidation, the slowest enzyme is again ME, thus accounting for the tendency for malate to escape before it can be converted to pyruvate.

**Table 2:** Kinetic data for ME, FumC, and AspA measured by conventional steady-state solution assays.

	Aspartate synthesis (downstream)			Aspartate oxidation (upstream)		
	ME	FumC	AspA	ME	FumC	AspA
*k*_cat_ (s^−1^)	17 ± 1.1	220 ± 54	503 ± 33	34 ± 3.1	220 ± 29	359 ± 15
*K*_M_ (mM)	8.6 ± 2.2	1.5 ± 0.11	0.19 ± 0.03	0.7 ± 0.22	0.6 ± 0.2	9 ± 1.6
*k*_cat/_*K*_m_(M^−1^ s^−1^)	2.0 × 10^3^	1.47 × 10^5^	2.65 × 10^6^	4.86 × 10^4^	3.67 × 10^5^	4.0 × 10^5^
*τ* (1/*k*_cat_, ms)	58	4.5	2	29	4.5	2.7

Numbers refer to mean values (*N* = 3 independent replicates) followed by standard errors.

### Comparing electrode nanoconfinement with conventional solution kinetics

Experiments were carried out to draw practical and fundamental comparisons with a non-confined cascade—an obvious difficulty being in comparing metrics between 3D homogeneous kinetics in solution and (at the primary macroscopic level) 1D heterogeneous kinetics with an electrode modified with buried enzymes, the local concentrations and accessibility of which are unknown (Fig. 1). Results are analyzed in Supplementary Information, Supplementary Table S1, and Supplementary Fig. S13. The rotating disc experiment shown in Fig. 3, which yielded an aspartate oxidation current of approximately 40 μA cm^−2^, corresponding (via current density/2 F) to a rate of 0.21 nmol cm^−2^ s^−1^ had been carried out after applying to the ITO/PGE electrode a 10 μL droplet containing 0.1 mM FNR, 0.5 mM ME, 0.1 mM FumC and 0.1 mM AspA. An electrochemical experiment with a different enzyme ratio was performed by applying a 20 μL droplet of enzymes (0.5 mM FNR and 0.1 mM in each of ME, FumC, and AspA) to the surface of an ITO/Ti electrode (area 1 cm^2^), incubating for 20 min then rinsing and placing in a cell containing 20 μM NADP^+^ and 20 mM aspartate at pH 7.5. This experiment, conducted with a larger stationary electrode, produced an aspartate oxidation current density of 28 μA cm^−1^ corresponding to a rate of 0.15 nmol cm^−2^ s^−1^.

The solution experiments were carried out using different dilutions of each 20 μL droplet into a cuvette containing 1 mL total buffer solution volume. The role of the electrode was now played by benzyl viologen, the reduction of which was monitored at 600 nm after initiating the reaction by injecting aspartate, to 20 mM, under anaerobic conditions. At a concentration (µM, in each of FNR/ME/FumC/AspA) of 4.2/0.85/0.85/0.85, the reaction rate corresponded to a rate of 0.9 mol of product per four-enzyme cascade unit per second. Successive dilutions (10-fold, 20-fold, 50-fold) resulted most obviously in greatly extended lag periods preceding slower rates.

On a practical level, the solution rates appeared far higher than the electrochemical rates when comparing on a mol s^−1^ (solution) to mol cm^−2^s^−1^ (electrode) basis, and suggested that a dilution of ~2000-fold (Supplementary Table 1) was required to give comparable rates. However, at a more fundamental level, two further factors must be considered. First, the uptake of all enzymes in the droplet into the ITO porous layer would result in an impossibly high enzyme concentration—at least 53 mM in total, assuming a layer of a maximum thickness (3 μm) and zero volume taken up by ITO itself. Based on the mass of a minimal catalytic unit, such a solution would weigh 18 kg L^−1^. While much lower concentrations of the enzyme can be loaded from solution, the adsorption times are much longer, so the concentrated droplet presented a rapid, albeit inefficient remedy. Second, the electrochemical kinetics of porous electrodes are complex: steady-state electrocatalysis will occur predominantly where enzymes are bound in regions closest to the solution boundary surface (Fig. 1b), leaving enzymes that are too buried essentially redundant^39,40^. Unlike a scaffold-supported cascade suspended in solution (Fig. 1a), which will be serviced by efficient 3-dimensional diffusion of reactants and escape of intermediates and products, diffusion to a densely-packed porous electrode surface is 1-dimensional. In an earlier study that proved the importance of nanoconfinement in a two-enzyme cascade, we proposed using a target area approximation—a 10 × 10 nm square of a hypothetically flat electrode to represent the capture activity of each catalytically competent nanoconfined cascade unit^35^. Using the same approach, the catalytic currents that we observed in the two representative electrochemical experiments (28 and 40 μA cm^−2^) equate to turnover frequencies (aspartate molecules produced per target area unit) of 90 and 126 s^−1^, respectively. For comparison, in the solution experiment, a turnover rate of ~1 s^−1^ (moles aspartate produced/moles of cascade unit) was measured for the fastest experiment.

A compelling piece of qualitative evidence overriding these metric difficulties concerns the initial lag period, so characteristic of enzyme cascade reactions measured conventionally in solution^41^. A lag is absent for the electrochemical experiment (a close-up of the region of Fig. 3d following aspartate injection is included in Supplementary Fig. S13) but is pronounced for the solution experiments and can even be seen for the most concentrated condition. As mentioned earlier, nanoconfinement traps intermediates, delaying their escape.

A Fermi-style approximation is instructive. Assuming a diffusion coefficient of 10^−5^ cm^2^ s^−1^ and a 1-dimensional free path to bulk solvent, an intermediate formed even at a depth of 1–2 μm should escape on a 1 ms timescale (in the crowded environment of the pore, this may be 3–4 times slower than in pure water^3^). The data in Table 2 can be used to obtain approximate *τ*-values (1/*k*_cat_^app^) that are relevant at the highest concentrations of intermediates. Whereas *τ*-values for FumC and AspA are ~5 and 2 ms, respectively, in either direction, quite close to the escape times, the average *τ*-value for ME is much higher, at 40 ms, making escape of the reactant more likely than its catalytic transformation. The high *τ*-value for ME suggests the importance of decreasing the probability of escape of an intermediate that is converted at a lower rate. The product and intermediate distribution correlate with individual enzyme activity rather than the practical catalytic potency (*k*_cat_^app^/*K*_M_^app^ × enzyme concentration applied): hence, the actual pore concentration of ME would need to be much higher than the other enzymes in order that conversion of malate to pyruvate competes with its escape.

### Advantages of electrode nanoconfinement for studying cascades

Some clear and objective comparisons can be drawn with the numerous studies of enzyme cascades immobilized on scaffolds that are usually freely dispersed in solution. First, the more efficient 3-dimensional (radial) diffusion geometry for the dispersed system (Fig. 1a) will enhance the supply of reactants and release of products, an unfortunate consequence being that intermediates escape more easily. Nonetheless, for dispersed systems, it is relatively straightforward to extend established enzyme kinetic models to address systematic influences of nanoconfinement, in particular, of substrate channeling^8,11,15–18,42^. Second, and in various systematic bottom-up approaches, the scaffolds and attached enzymes can be pre-assembled to give structures with known enzyme composition and intersite distances: such an ideal situation is difficult to achieve in pre-deposited tight nanopores, where enzyme cascade composition and intersite distances are currently probabilistic. Third, the electrochemical system, in which FNR (E1) works as an in-situ transducer has no counterpart in the dispersed system; not only are rates observed directly and in real-time (Fig. 1b), but the reaction direction can be switched back and forth, as we have stated above, via a laptop command to the potentiostat. The ability to regulate how fast E1 works in either direction even offers the possibility to alter the rate-determining step.

The experiments prove the ability to energize, control, and observe, in real-time, long cascades under nanoconfinement where local enzyme concentrations might also be extremely high and reaction partners crowded, a condition prevailing in zones of living cells. While much needs to be done to understand and optimize the loading and binding of enzymes in the material (particularly, confining them to the more shallow pores), it is difficult to see how such an advantageous situation could be obtained in another way. The variety and abundance of NAD(P)H-linked dehydrogenases that are now available afford unlimited scope for coupling, via extended cascades, to myriad enzymes of all types, driving and detecting the catalytic chemical flow that results via the transducer FNR. In fundamental explorations, the technology allows rapid optimization of reaction conditions, particularly for cascades that are driven upstream. The nanoconfined and energized cascade allows real-time, interactive kinetic measurements of the behavior of concentrated (and possibly crowded) multi-enzyme systems as opposed to individual enzymes: a notable application is the possible spontaneous assembly of active enzyme complexes such as metabolons, and our approach may be unique in its potential to shed light on this important area^2,6,7^. Such a simple concept and methodology should be very valuable in many aspects of biocatalysis, ranging from synthesis to sensing, new enzyme discovery, and even time-resolved activation and inactivation of enzymes lacking a useful spectroscopic handle: indeed, an indefinite number of possibilities emerge for building and connecting bespoke cascades.

## Methods

### Ferredoxin NADP^+^ reductase (FNR): purification

A vector (aLICator pLATE51, N-terminal His-tag/EK, #K1251, Thermo Scientific,) containing the gene encoding FNR from *Chlamydomonas reinhardtii* with an N-terminal hexa-Histag was used to transform *Escherichia coli (E. coli) cells* (BL21) (New England Biolabs). Positive colonies were selected by resistance to ampicillin, conferred by the gene encoding β-lactamase (bla(Ap^R^)) present in the vector. Approximately 100 mL of Luria-Bertani (LB) medium supplemented with ampicillin (final concentration, 0.27 mM) was inoculated with one positive colony and grown overnight at 37 °C in a shaking incubator (200 rpm). This culture was then diluted (~10 mL into 500 mL; typically 6 × 500 mL per FNR preparation) into the same medium (LB containing 0.27 mM ampicillin) and grown (37 °C, 200 rpm) until the cells reached the mid-log phase at which point they were induced by addition of isopropyl β-d-1-thiogalactopyranoside (IPTG) to a final concentration of 1 mM and grown for a further 3–4 h. The cells were then harvested by centrifugation (8983 × *g*) at 4 °C for 30 min and the pellets resuspended in cold cell resuspension buffer (50 mM HEPES; 150 mM NaCl; 10% V/V Glycerol, pH 7.4) and stored at −80 °C until purification. The resuspended cells were thawed and then lysed using a French press at 20 psi. Insoluble material was removed by centrifugation at 208,429 × *g*, 4 °C for 1 h, and purification of FNR in the resulting supernatant was carried out using an ÄKTA system for nickel affinity chromatography (Ni^2+^ HisTrap HP affinity column, GE Healthcare Life Sciences) using buffer A (for pre-equilibration and washing of the column) consisting of 50 mM HEPES, 0.5 M NaCl, 1 mM dithiothreitol (DTT), pH 7.4, and elution buffer B (50 mM HEPES, 0.5 M NaCl, 0.25 M imidazole, 1 mM DTT, pH 7.4). The FNR was eluted in 1 mL aliquots using a linear imidazole gradient reaching 0.25 M imidazole over ~40 mL. Fractions containing FNR were selected based on the absorbance at 280 nm and 460 nm, pooled, then concentrated using Amicon® Ultra 4 mL centrifugal 10 kDa filters to a final volume of ~2 mL. The concentrated protein was passed through a desalting column (PD-10 GE Healthcare) to remove imidazole, portioned into single-use aliquots, flash-frozen in liquid nitrogen, and stored at −80 °C. Enzyme concentration was determined by the Bradford assay^43^.

### Malic enzyme, l-malate-NADP^+^ oxidoreductase (decarboxylating): purification

A vector (aLICator pLATE51, N-terminal His-tag/EK, #K1251, Thermo Scientific) containing the MaeB gene (encoding malic enzyme from *E. coli* with an N-terminal hexa-Histag) was used to transform *E. coli* cells (BL21) cells (New England Biolabs). A single colony was used to inoculate 100 mL of LB medium supplemented with ampicillin (final concentration, 0.27 mM) and grown at 37 °C in a shaking incubator (200 rpm) for 16 h after which time it was diluted (15 mL into 500 mL; typically 12 × 500 mL per malic enzyme preparation) of the same medium and grown for a further 3.5 h (37 °C, 200 rpm). At this point, the cells were induced by the addition of IPTG to a final concentration of 0.5 mM followed by growth at 15 °C for 20 h. The cells were harvested as described for FNR and the pellets resuspended in buffer (pH 7.4) containing 50 mM HEPES, 150 mM NaCl, 10% glycerol, and EDTA-free protease inhibitors (Roche). The cell suspension was frozen at −80 °C until purification. Purification was carried out as described above for FNR with the following differences: Buffer A (50 mM HEPES, 0.50 M NaCl, 50 mM imidazole, 1 mM DTT, pH 7.4) and elution buffer (50 mM HEPES, 0.50 M NaCl, 0.30 M imidazole, 1 mM DTT, pH 7.4). The protein was eluted using a linear imidazole gradient. Positive fractions were selected based on a malic enzyme activity assay. Briefly, the oxidation of malate (50 mM) was monitored by the change in absorbance at 340 nm resulting from the concomitant reduction of NADP^+^ (1 mM), using a UV/Vis spectrophotometer (Perkin Elmer, Lambda 19). Active fractions were pooled and the malic enzyme was concentrated in centrifuge tubes with a 50 kDa selective membrane (Amicon®) and dialyzed overnight at 4 °C against buffer containing 50 mM HEPES, 150 mM NaCl, 20 mM MnCl_2_, 1 mM DTT, pH 7.4, and 10% glycerol. Protein aliquots (20 µL) were flash-frozen in liquid nitrogen and stored at −80 °C. Enzyme concentration was determined using the Bradford assay^43^.

### l-Aspartate ammonia-lyase: cloning, expression, and purification

The following primers (AspA Forward and AspA Reverse, see Supplementary Table 2) were designed based on the sequence reported in KEGG (entry b4139). After confirming successful amplification, the PCR product was purified and ligation-independent cloning was performed (aLICator expression system, Thermo Fisher) using a pLATE51 vector, to add an N-terminal hexa-HIStag (as per the manufacturer’s instructions). DH5-α cells (New England BioLabs, NEB) were transformed as in the manufacturer’s protocol and grown overnight at 37 °C on an LB/agar plate supplemented with ampicillin to a final concentration of 0.27 mM. A single colony was then used to inoculate 5 mL of LB supplemented with ampicillin (final concentration, 0.27 mM) and grown at 37 °C for 16 h. The cell suspension was centrifuged at 3213 × *g* for 10 min, the medium discarded, and plasmid DNA isolated using a miniprep kit (Qiagen). Sequence verification was carried out by Source BioScience (University of Oxford). The new vector (pLATE51:aspA) was used to transform *E. coli* BL21 cells (NEB) as per the manufacturer’s instructions and a single colony was used to inoculate 100 mL LB (containing ampicillin at 0.27 mM) and grown overnight at 37 °C and 250 rpm. 15 mL of this culture was then diluted into 500 mL LB (supplemented with ampicillin to 0.27 mM) and grown for a further 3 h. The cells were induced by the addition of IPTG (1 mM) and expression was allowed to continue for 20 h at 20 °C. Cells were harvested at 8983 × *g* for 30 min at 4 °C and resuspended in a solution consisting of 50 mM HEPES, 150 mM NaCl, 10% glycerol supplemented with two tablets of EDTA-free protease inhibitors (Roche); the cell suspension was then frozen at −80 °C until purification.

The enzyme was purified as described for FNR with the following changes: buffer A (50 mM HEPES, 500 mM NaCl, 50 mM imidazole, 1 mM DTT, pH 7.4); buffer B (50 mM HEPES, 500 mM NaCl, 300 mM imidazole, 1 mM DTT, pH 7.4). The protein was eluted over a linear imidazole gradient and the relevant fractions were selected by monitoring the absorbance at 280 nm and also verified by an activity solution assay (described later). The protein was then concentrated to 1 mL using a centrifugal tube with a 50 kDa membrane (Amicon®) and dialyzed overnight against 1 L of dialysis buffer (50 mM HEPES, 150 mM NaCl 10% glycerol, pH 7.4). Single-use aliquots of the enzyme were flash-frozen in liquid N_2_ and stored at −80 °C.

### Fumarase (fumarate hydratase): purification

Expression and purification of FumC through pASK40:*fumC* was performed using a protocol adapted from Jones and Hirst^44^. We are grateful to Professor Todd Weaver, University of Wisconsin–La Crosse, for constructing the plasmid^45^. Briefly, expression and purification were carried as for l- aspartate ammonia-lyase with the following changes: (i) before IPTG induction, the bacterial culture was grown at 32 °C until reaching an OD (*A*_600 nm_) of 0.6, (ii) after harvesting, the cells were resuspended in buffer containing 50 mM HEPES, 300 mM NaCl, and two tablets of EDTA-free protease inhibitors (pH 7.8), (iii) buffer A contained 50 mM HEPES, 0.30 M NaCl, 60 mM imidazole, 1 mM DTT, (pH 7.8), (iv) buffer B contained 50 mM HEPES, 0.30 M NaCl, 400 mM imidazole, 1 mM DTT, (pH 7.8), and (v) dialysis was carried out with a buffer containing 50 mM HEPES, 0.15 M NaCl, 10% glycerol, pH 7.8.

### Carbonic anhydrase (carbonate dehydratase)

Lyophilized bovine carbonic anhydrase (Sigma) was dissolved in 50 mM HEPES, pH 7.5 for subsequent use in the reduction direction and 50 mM MOPS, pH 7.5 for use in the oxidation direction.

### Preparation of electrodes

A piece of titanium foil (Sigma, 0.127 mm thick, 99.7%) cut to size and mounted on a connecting support was cleaned by successive (15 min) sonications in deionized water (Milli-Q^TM^, 18 MΩ cm), ethanol, and acetone. Indium tin oxide (ITO) nanoparticles (Sigma, <50 nm) were then deposited by electrophoretic deposition as described previously^33^. Briefly, a solution of I_2_ (0.1 g) and ITO (0.2 g) was prepared in acetone (20 mL) and a potential of 10 V applied for 6 min using a second piece of Ti foil (of equal size or larger) as auxiliary electrode held ~1 cm away. The electrodes were rinsed thoroughly before loading the enzymes. Analogous procedures were used to prepare ITO/PGE electrodes for rotating disc experiments^33^.

Enzymes were mixed at specific ratios (CA/FNR/ME/FumC/AspA) and then 20 μL of the enzyme solution was deposited and spread on the ITO layer (20 μL per approximate 1.9 cm^2^); this was altered to 10 μL for the ITO/PGE electrode. The electrode was then left at room temperature for 20 min before rinsing thoroughly in ultrapure water to remove any unbound enzyme.

### Electrochemical measurements

A three-compartment cell was used for all cyclic voltammetry and chronoamperometry experiments (except for the rotation experiments), using a Pt mesh as a counter electrode, and an Ag/AgCl reference electrode. Experiments were carried out using PalmSens multi-channel or Metrohm Autolab (PGSTAT128N) potentiostats. The potentials were converted to SHE using the following equation: *E*_SHE_ = *E*_Ag/AgCl_ + 0.21 V. The programs PSTrace 4.8 and MultiTrace were used in all electrochemical experiments unless stated otherwise. Cyclic voltammetry (pH 7.5, 25 °C) was carried out using a buffer containing 20 mM sodium pyruvate (Sigma, >99%), 0.1 M NH_4_Cl, 0.1 M KHCO_3_, 4 mM MgCl_2_, and 1 mM MnCl_2_. An ITO/Ti electrode (1.98 cm^2^) was prepared as described previously and a droplet of the enzyme mixture in the required ratios and concentrations was deposited on the electrode and kept for 20 min at room temperature to allow the enzymes to adsorb. The electrode was then thoroughly rinsed with deionized water. Throughout the experiment, the cell was continuously flushed with Argon.

Chronoamperometry experiments were carried out to drive the downstream reductive amination/carboxylation of pyruvate to aspartate or the upstream oxidative deamination/decarboxylation of aspartate to pyruvate. In the former case, a potential of −0.45 V vs SHE was applied. Experiments were carried out in an anaerobic glove box (MBraun) with an N_2_ atmosphere (O_2_ < 2 ppm). The surface area for aspartate synthesis (the downstream direction) was approximately 12 cm^2^. The buffer used was 0.05 M HEPES, 0.1 M NH_4_Cl, 4 mM MgCl_2_, and 1 mM MnCl_2_, 20 mM pyruvate (pH 7.5). The cell solution was stirred using a small magnetic stirrer bar. Reactions were initiated by injection of NADP^+^ (to a final concentration of 20 μM) and KHCO_3_ (to a final concentration of 0.1 M). Aspartate oxidation (the upstream direction) was carried out on the bench under a continuous flow of Argon, using an electrode surface area of typically 3.6 cm^2^. NADP^+^ was added to a final concentration of 20 μM and potassium l-aspartate (Sigma, 98.5%) to 20 mM in a buffer containing 0.05 M MOPS (Melford), 4 mM MgCl_2_, and 1 mM MnCl_2_, pH 7.5.

Rotating disc electrode experiments were carried out in an anaerobic glove box (MBraun) with an N_2_ atmosphere (O_2_ < 2 ppm) to ensure that there was no contribution to the current by O_2_ upon injection of substrates. An ITO/PGE electrode (0.03 cm^2^) was loaded with a mixture of the enzymes in the following proportions (0.1/1/5/1/1, CA/FNR/ME/FumC/AspA) by drop-casting 10 μL of the enzyme mixture and incubating at RT for 20 min. The electrode was then thoroughly rinsed to remove any unbound protein. The reference electrode (Ag/AgCl) was housed in a sidearm and connected to the main cell via a Luggin capillary; the Pt counter electrode was present in the same compartment as the working electrode. Nova version 1.10.4 (Metrohm) was used to collect the data. The buffer used was 0.05 M HEPES, 0.1 M KHCO_3_, 0.1 M NH_4_Cl, 20 mM pyruvate, 4 mM MgCl_2_, 1 mM MnCl_2_ at pH 7.5. An aliquot of concentrated NADP^+^ solution was injected to give a final cell concentration of 20 μM. The rotation rate (*ω*) at the beginning of the experiment was 1000 rpm and the potential applied was −0.45 V vs SHE at 25 °C. The rotation rate was switched back and forth between 0 and 1000 rpm. After 1 h the cell solution was removed, the electrode was placed in a buffer, and the compartment washed 10 times with deionized water and once with buffer. A new buffer containing 0.05 M MOPS, 4 mM MgCl_2_, 1 mM MnCl_2_ at pH 7.5 was added and the electrode reinserted into this fresh cell solution. The potential was then changed to 0.1 V SHE and rotation resumed (*ω* = 1000 rpm). Injection of aspartate to 20 mM caused the immediate (within 2 s) appearance of an oxidation current. Again, the rotation was switched between 1000 and 0 rpm.

### ^1^H-NMR spectroscopy

A Bruker AVIIIHD 400 instrument was used to measure NMR spectra. For quantification, an internal standard, 3-(trimethylsilyl)propionic-2,2,3,3-d_4_ acid sodium salt (TMSP—4.2 mM), was used. The NMR spectra were visualized using MestreNova version 11.03. Prior to NMR, the samples (in 10% D_2_O) were treated by the addition of EDTA (pH 8) to a final concentration of 10 mM in order to sequester paramagnetic Mn^2+^.

### Determination of kinetic parameters for l-malate-NADP^+^ oxidoreductase

A UV-vis spectrophotometer (Perkin Elmer, Lambda 19). was used to investigate the solution kinetic parameters of the enzymes. The buffer used for the reductive carboxylation was 0.05 M HEPES, 0.1 M NH_4_Cl, 4 mM MgCl_2_, 1 mM MnCl_2_ pre-saturated with high purity CO_2_ for 4 h. An aliquot of concentrated NADPH solution was added to give a final cell concentration of 0.2 mM and the consumption of NADPH monitored through the decrease in absorbance at 340 nm using an absorption coefficient of 6.22 mM^−1^ cm^−1^. Pyruvate was added to give final concentrations of 0, 1, 2, 4, 6, 8, 10, 20, 50 mM and triplicates of each measurement were performed. Measurements were carried out using an Ocean Optics S2000 fiber optic spectrometer controlled with OOIBase32 software.The data were first imported in Excel and then fitted using Origin Pro 2020.

The buffer used for oxidative decarboxylation was 0.05 M MOPS, 4 mM MgCl_2_, 1 mM MnCl_2_ (pH 7.5); NADP^+^ was added to a final concentration of 1 mM and different malate concentrations were tested (0, 0.05, 0.1, 0.5, 1, 2, 4, 6, 8, 10, 20, 50 mM). The production of NADPH was monitored as the increase in absorbance at 340 nm. Data were fitted using Origin Pro 2020.

### Determination of kinetic parameters for fumarase and l-aspartate ammonia-lyase

For the hydration of fumarate to malate and amination of fumarate to aspartate, the buffer used was 0.05 M HEPES, 0.1 M NH_4_Cl, 4 mM MgCl_2_, 1 mM MnCl_2_. Fumarate consumption was monitored using the changes in absorbance at 250 nm (from 0 to 0.9 mM, absorption coefficient 1.45 mM^−1^ cm^−1^) and 290 nm (from 1 mM to 20 mM, absorption coefficient 0.082 mM^−1^ cm^−1^).

For the dehydration of malate to fumarate and deamination of aspartate to fumarate, the buffer used was 0.05 M MOPS, 4 mM MgCl_2_, 1 mM MnCl_2_ (pH 7.5), and fumarate formation was monitored as the change in absorbance at 250 nm. Concentrations tested were: for malate; 0, 0.05, 0.1, 0.5, 1, 2, 4, 6, 8, 10, 20, 50 mM; for aspartate, 0.02, 0.05, 0.1, 0.5, 1, 2, 4, 8, 10, 20, 50 mM.

### Comparing rates of the unconfined cascade in solution vs nanoconfined cascade in electrode

Activities of the unconfined cascade at various concentrations in a solution volume of 1 mL were measured at room temperature by monitoring the reduction of benzyl viologen at 600 nm. All reactions were carried out in an anaerobic glove box (Belle Technologies) with an N_2_ atmosphere (O_2_ < 2 ppm) under stirred conditions. Enzymes, benzyl viologen (50 mM), and NADP^+^ (20 µM) were present from the start and the reaction was initiated by the addition of aspartic acid to a final concentration of 20 mM. The buffer used was 50 mM MOPS, pH 7.5 (adjusted using KOH), supplemented with MgCl_2_ (4 mM) and MnCl_2_ (1 mM).

### Reproducibility

All experiments were repeated independently in triplicate unless stated otherwise.

### Reporting summary

Further information on research design is available in the Nature Research Reporting Summary linked to this article.

## Supplementary information

Supplementary Information

Reporting Summary

## Data Availability

All data are available on request from the authors. The primers to amplify AspA were designed based on the nucleotide sequence available at www.genome.jp/dbget-bin/www_bget?eco:b4139.
