# Supplementary material for: The power of electrified nanoconfinement for energising, controlling and observing long enzyme cascades
Source: Nat Commun. 2021 Jan 12;12:340. doi: 10.1038/s41467-020-20403-w (PMC7804111; doi:10.1038/s41467-020-20403-w)
Supplement: Supplementary file 1 — Supplementary Information [file 41467_2020_20403_MOESM1_ESM.pdf]

Supplementary Information

The power of electrified nanoconfinement for energising, controlling and observing long enzyme cascades

Giorgio Morello, Clare F. Megarity and Fraser A. Armstrong *

Inorganic Chemistry Laboratory, Department of Chemistry, University of Oxford, South Parks road, Oxford, OX13QR

*email: fraser.armstrong@chem.ox.ac.uk

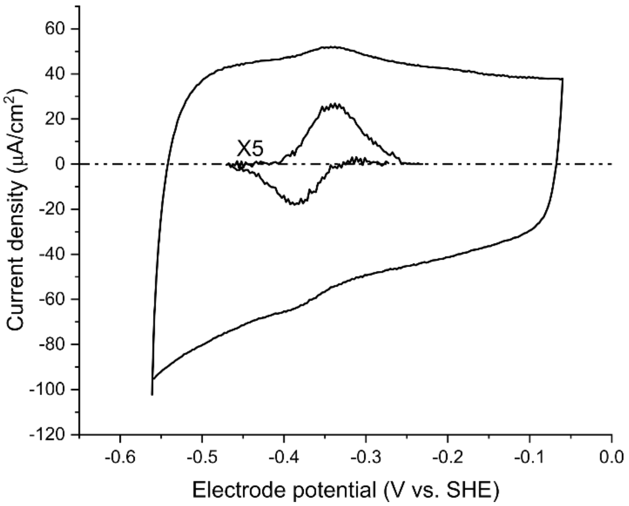

Supplementary Figure 1. Calculation of FNR coverage from non-turnover peaks. By integrating the charge passed (coulombs) under each of the non-turnover peaks (and taking an average) it is possible to determine the amount of electroactive FNR absorbed on the electrode using the relationship - Quantity (in moles) = *charge*/*nF*, where *n* is the number of electrons involved (2) and *F* is the Faraday constant.

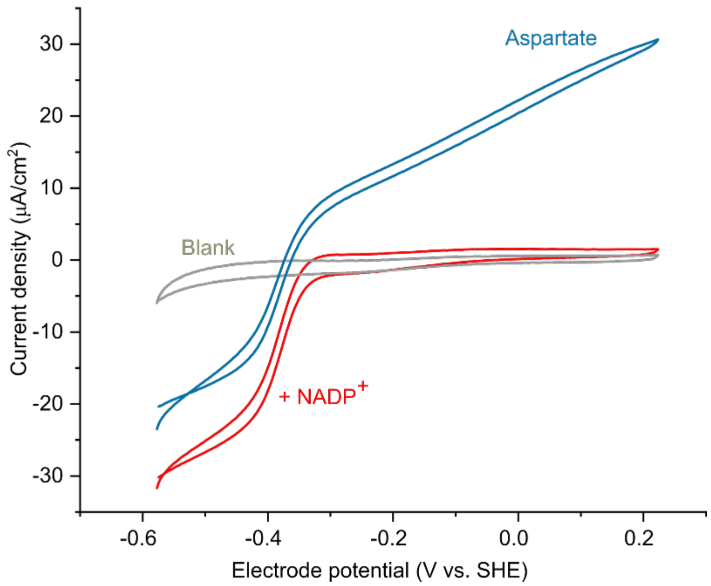

Supplementary Figure 2. Cyclic voltammograms of the 5-enzyme cascade without any added buffer. Enzyme ratio in droplet applied to electrode: 0.1 CA/1 FNR/ 5 ME/1 FumC/ 1 AspA. Solution contained: 20 mM pyruvate, 0.1 M ammonium carbonate, 4mM $MgCl_2$, 1 mM $MnCl_2$, adjusted to pH 7.5. Scan rate 1 mV/s; 25°C. Grey: blank, no cofactor present. Red: after injection of $NADP^+$ (to 20 µM). Blue: after injection of L-aspartate (to 20 mM).

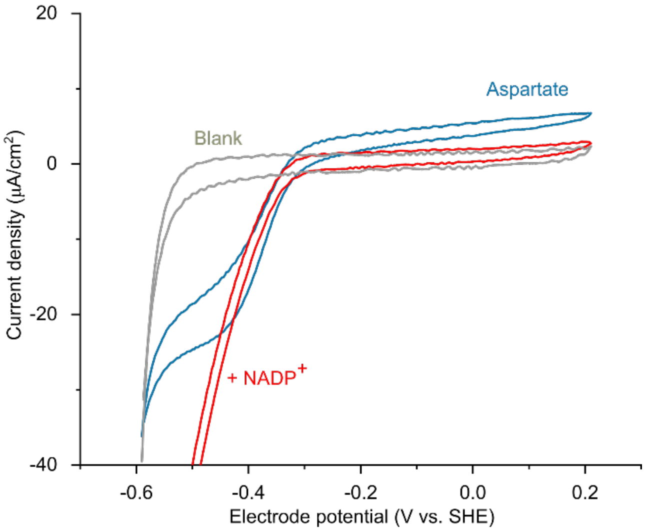

Supplementary Figure 3. Cyclic voltammograms of the 4-enzyme cascade in CO_2-saturated buffer. Enzyme ratio in droplet applied to the electrode: 1 FNR / 5 ME / 1 FumC / 1 AspA. Buffer: 0.2 M MOPS, 0.1 M $KHCO_3$, 4 mM $MgCl_2$, 1 mM $MnCl_2$, 20 mM pyruvate in 100% CO_2 (initial pH 7.5). Scan rate: 1 mV/s; 25°C. Grey: Blank, no cofactor present. Red: after injection of $NADP^+$ to a final concentration of 20 μM. Blue: after injecting L-aspartate to a final concentration of 20 mM.

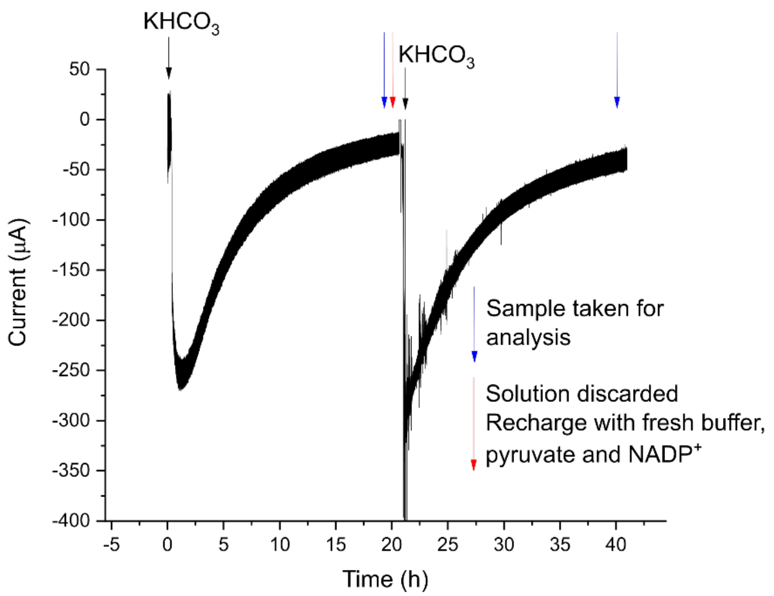

Supplementary Figure 4. Reductive amination/carboxylation of pyruvate to aspartate. Conditions: stirring, electrode potential = -0.45 V vs SHE, 25°C. Buffer: 0.05 M HEPES, 4 mM $MgCl_2$, 1 mM $MnCl_2$, 20 µM $NADP^+$, pH 7.5. A high surface area electrode (12 cm^2) was used to increase yield for detection of products by ^{1}H-NMR. The nanoporous indium tin oxide was loaded with the following enzyme ratio: 0.1 CA / 1 FNR / 2 ME /1 FumC / 1 AspA. Injection of $KHCO_3$ (to 0.1 M) triggered the start of the cascade reaction. The rate (reduction current) rapidly increased and remained stable for 5 hours after which it decreased over the course of 1 day. After 20 hours (blue arrow) the solution (4.5 mL) was tested by ^{1}H-NMR and contained 6.80 mM aspartate, 0.06 mM fumarate and 1.4 mM malate. Thus, a conversion of 34% to aspartate was obtained after 24 hours with a total turnover number (TTN, [aspartate]/[NADP+]) of 340. Replacement of the solution with fresh buffer and substrate reinjection resulted in the restoration of the original current. In the second 20 h a conversion of 24 % was obtained with a TTN of 240. The solution contained 4.8 mM aspartate, 0.02 mM fumarate and 1.26 mM malate.

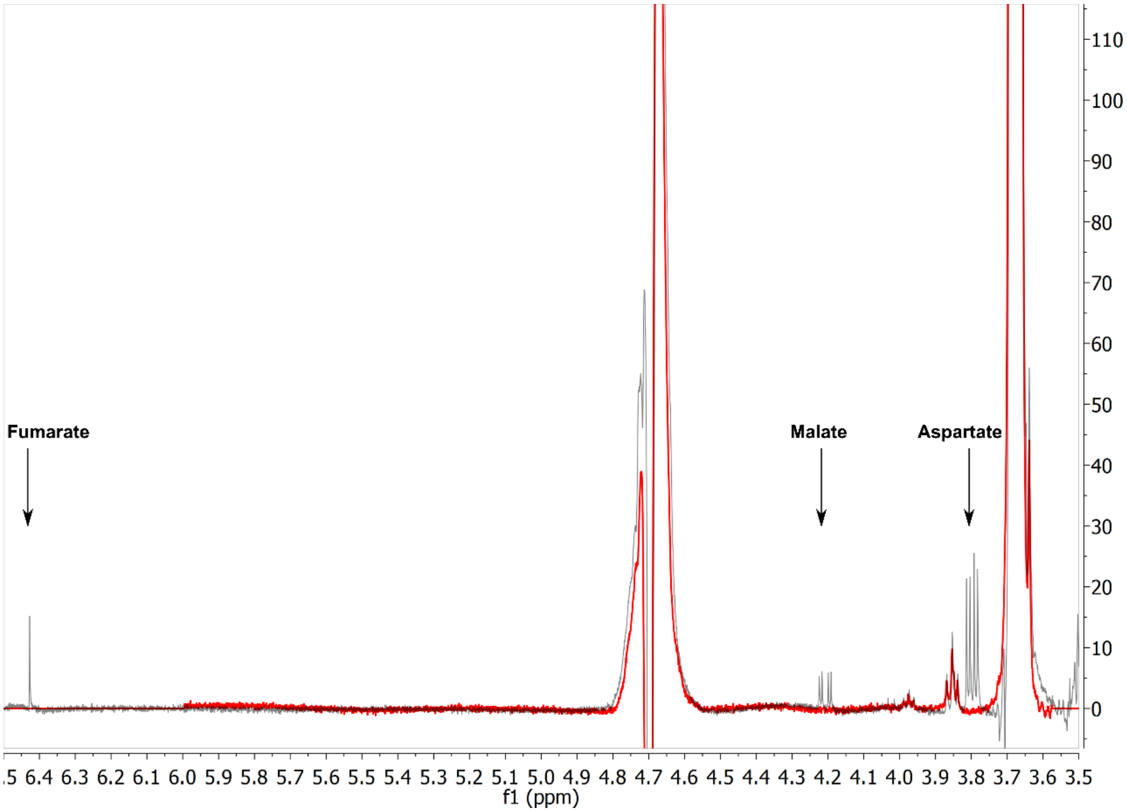

Supplementary Figure 5. Typical ^{1}H-NMR spectrum of the product mixture for the conversion of pyruvate to aspartate. Samples in 10% D_2O. Prior to analysis samples were treated with EDTA (final concentration 10 mM). Red: time 0. Grey: time 20 hours. Characteristic signals correspond to fumarate (6.4 ppm, singlet), malate (4.2 ppm, doublet of doublets) and aspartate (3.8 ppm, doublet of doublets).

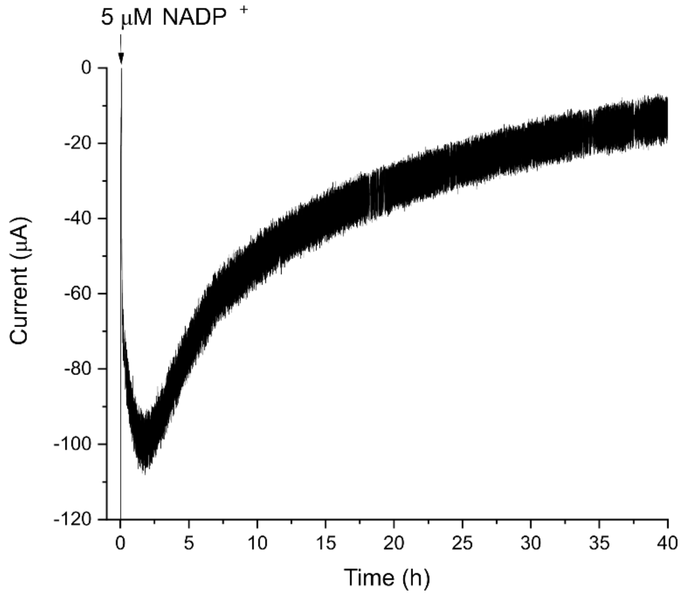

Supplementary Figure 6. Conversion of pyruvate to aspartate using lower (5 μM) $NADP^+$ concentration. Conditions: stirring, electrode potential = -0.45 V vs SHE, 25°C. Buffer used: 0.05 M HEPES, 0.1 M $KHCO_3$, 0.1 M NH_4Cl, 4 mM $MgCl_2$, 1mM $MnCl_2$, 20 mM pyruvate, 5 μM $NADP^+$, pH 7.5. Electrode surface area: 12 cm^2. Enzyme ratio in droplet applied to electrode: 0.1 CA / 1 FNR / 5 ME / 1 FumC / 1 AspA. After 40 h, 23.4% of pyruvate was converted to aspartate and the TTN ([aspartate]/[NADP+]) was 960. From NMR, quantities are: asp = 0.021 mmol, total (asp + fum + mal) = 0.025 mmol, while from coulometry, total amount = 0.029 mmol

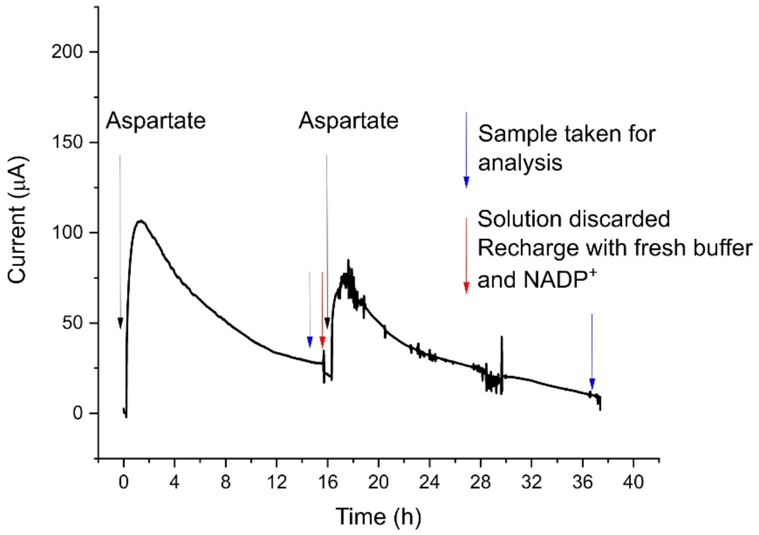

Supplementary Figure 7. Oxidative deamination/decarboxylation: conversion of L-aspartate to pyruvate. Conditions: stirring, electrode potential = +0.1 V vs SHE, 25°C. Buffer: 0.05 M MOPS, 4 mM $MgCl_2$, 1 mM $MnCl_2$, 20 μM NADP+, pH 7.5. Electrode surface area: 3.5 cm^2. The nanoporous indium tin oxide (ITO) electrode was loaded with a droplet having the following enzyme ratio: 0.1 CA / 1 FNR / 1 ME / 1 FumC / 1 AspA. Injection of L-aspartate (to 20 mM) triggered the start of the cascade reaction; the rate rapidly increased (oxidation current became more positive). After 16 hours (blue arrow) the solution (4 mL) was tested by ^{1}H-NMR. Stability of the confined enzyme cascade was confirmed since replacement of the solution with fresh buffer and recharging with substrate resulted in a rate close to the original value, and the same distribution of intermediates and product was maintained (Figure 4 in main paper).

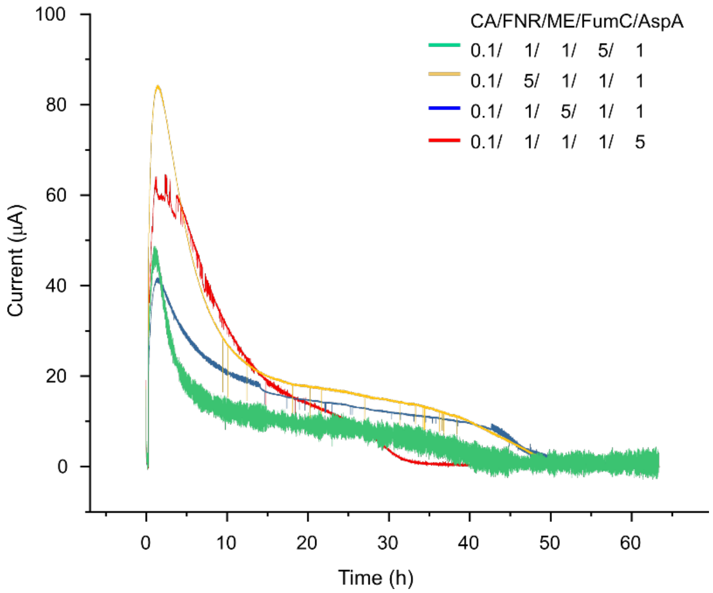

Supplementary Figure 8. Oxidative deamination/decarboxylation of aspartate to pyruvate: effect of changing relative ratio of enzymes in droplet applied to electrode. Conditions: stirring, electrode potential = +0.1 V vs SHE, 25°C. Buffer: 0.05 M MOPS, 4 mM $MgCl_2$, 1 mM $MnCl_2$, 20 μM $NADP^+$, pH 7.5. Electrode surface area: 2.89 cm^2. Enzyme ratios (CA/FNR/ME/FumC/AspA): red 0.1/1/1/1/5; blue 0.1/1/5/1/1; yellow 0.1/5/1/1/1; green 0.1/1/1/5/1. Reactions were initiated by injection of L-aspartate to a final concentration of 20 mM. The charges passed (integral of the current-time trace) correspond to the final amount of product formed.

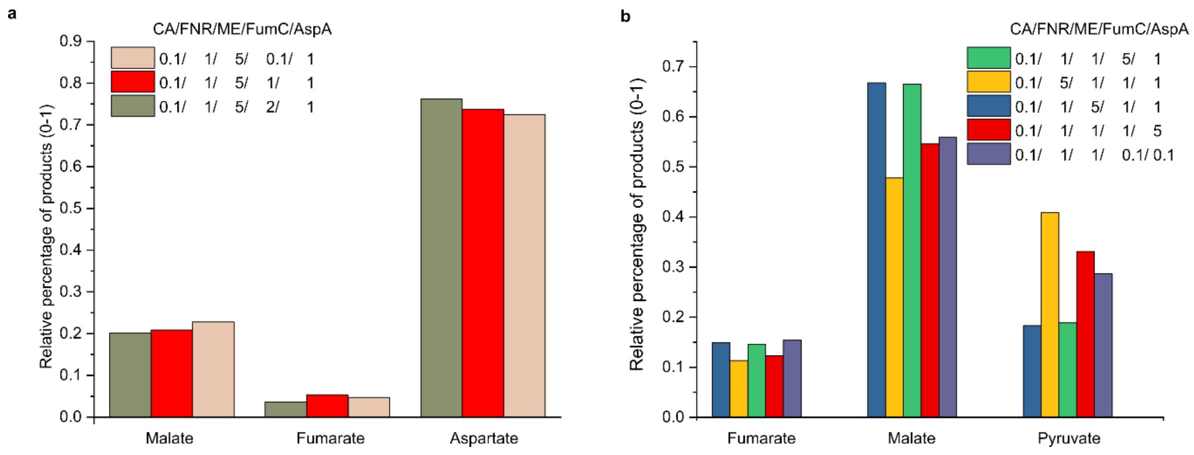

Supplementary Figure 9. Relative product distributions obtained upon changing the ratios of enzymes. Panel **a** shows results obtained for the downstream direction (pyruvate reduction) where fumarase ratio was varied from 0.1 to 2. Panel **b** shows results obtained for the upstream direction (aspartate oxidation). Each bar represents a single measurement.

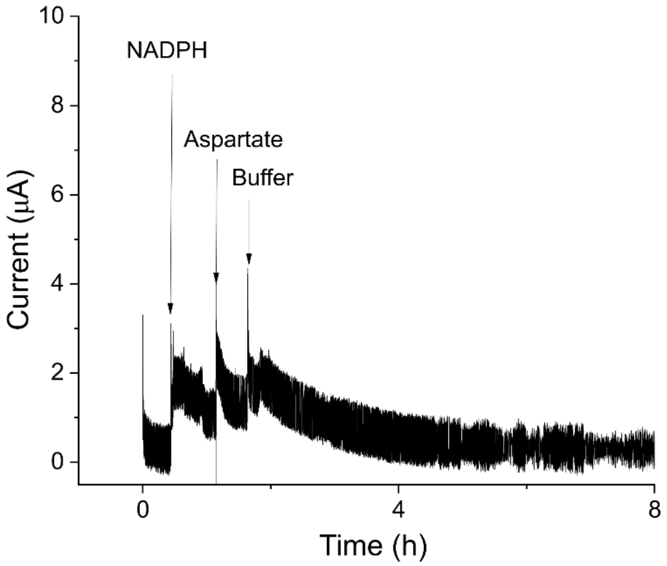

Supplementary Figure 10. Control experiment in which four enzymes were deposited on different areas of electrode. Each enzyme (FNR/ME/FumC/AspA) was dropped onto a different area of the electrode surface making sure that there was no contact between each spot of enzyme solution. Conditions: stirring, electrode potential = +0.1 V vs SHE, 25°C. Electrode surface area 1 cm^2. Enzymes added were FNR, ME, FumC and AspA. The experiment proves that nanoconfinement of all enzymes together in the nanopores is essential for activity. Buffer: 0.05 M MOPS, 4 mM $MgCl_2$, 1 mM $MnCl_2$, pH 7.5. NADPH was injected to a final concentration of 20 μM and aspartate to a final concentration of 20 mM. The last injection of buffer was a control step. No catalysis was observed upon aspartate injection.

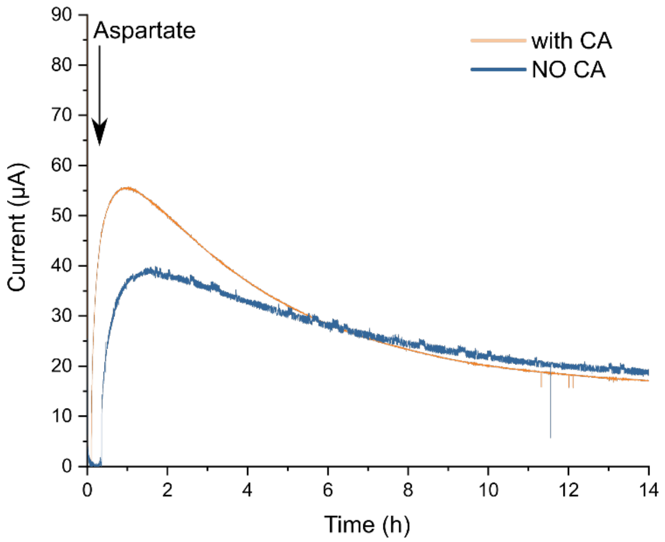

Supplementary Figure 11. Comparison of the upstream reaction (oxidation of aspartate) with or without carbonic anhydrase (CA). Conditions: stirring, electrode potential = +0.1 V vs SHE, 25°C. Electrode surface area 2.9 cm^2. Buffer: 0.05 M MOPS, 4 mM $MgCl_2$, 1 mM $MnCl_2$, 20 µM $NADP^+$ pH 7.5. Enzyme ratio: 0.1 CA/1 FNR/1 ME/ 1 FumC/1 AspA. Arrow indicates the injection of 20 mM L-aspartate. The absence of CA does not affect the upstream reaction.

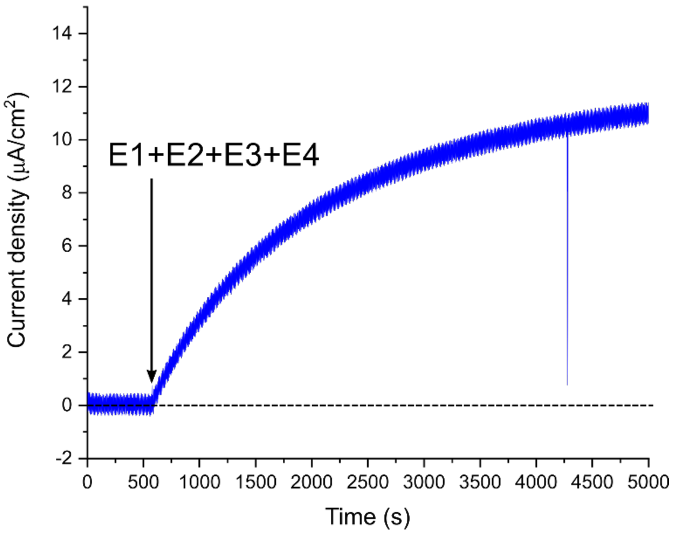

Supplementary Figure 12. Control experiment showing that aspartate is not electroactive at the potential applied and injection of E1 to E4 initiates catalysis. Conditions: stirring, electrode potential = +0.1 V vs SHE, 25°C. Electrode surface area 2.9 cm^2. Buffer: 0.05 M MOPS, 50 mM L-aspartate, 4 mM $MgCl_2$, 20 mM $MnCl_2$, 20 µM $NADP^+$, pH 7.5. Arrow indicates injection of E1 to E4 at a ratio of 1 FNR / 5 ME / 1 FumC / 1 AspA at a final concentration of 0.5 µM in the case of FNR, FumC and AspA, while ME was injected to a final concentration of 2.5 µM.

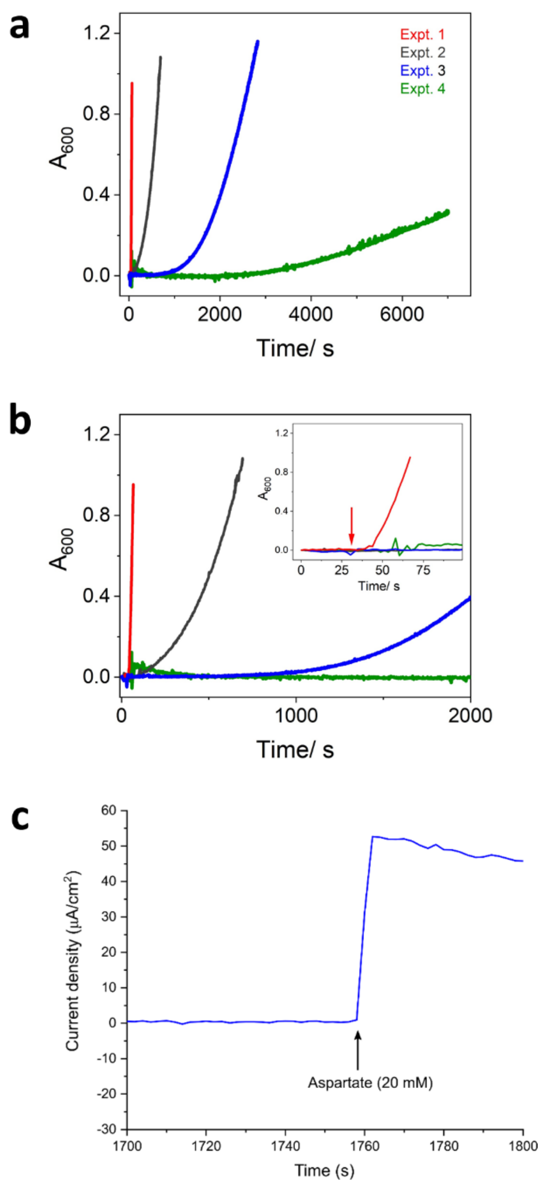

Supplementary Figure 13. Cascade activity in solution. **a:** Full view. **b**: Truncated view to show the lag; inset: magnification of the start of experiment 1 (red). Benzyl viologen (50 mM) was used as a reporter and mediator for cofactor recycling by FNR; activity monitored using the absorbance at 600 nm due to reduced benzyl viologen. 20 μM $NADP^+$, 20 mM aspartic acid, activity initiated by the addition of aspartic acid. **c:** Magnification of the initiation by aspartic acid addition in the electrochemistry experiment shown in Figure 3d, note the immediacy and absence of a lag. For a and b: experiment 1 was a single measurement; experiment 2 was performed in duplicate with the reaction initiated by addition of aspartate; experiment 3 was a single measurement; experiment 4 was performed in duplicate.

Supplementary Table 1: A comparison of the cascade monitored free in solution vs nanoconfined in a porous electrode

	Expt.	Final concentration of each enzyme in cuvette (nM)				Total amount of cascade in cuvette (nmoles)	Dilution factor from [cascade] in 20 µl droplet used in electrochemistry expt. 5 to give the [cascade] in each solution expt.	Rate (nmoles s^{-1})	Rate as moles product per total moles of enzyme (s^{-1}) *rate still increasing	Hypothetical [Cascade] in a porous electrode of 1cm X 1cm X 3um (mM)	
		FNR	ME	FumC	AspA						
Mediated by Benzyl Viologen (1 mL)	1	4160	851	857	853	6.72	119	(5.98)	0.89	22.4	
	2	416	85	85	85	0.672	1192	(0.421)	0.63*	2.24	
	3	208	42.5	42.8	42.6	0.336	2381	(0.138)	0.41*	1.12	
	4	83	17	17	17	0.134	5970	(0.0135)	0.10*	0.45	
		**Final Concentration of each enzyme in droplet (mM)**				**Total amount of cascade in droplet (nmoles)**		**Rate (nmoles $cm^{-2}s^{-1}$)**	**Rate as moles product per total moles of enzyme (s^{-1})**	**Hypothetical [Cascade] in electrode of 1cm X 1cm X 3µm (mM)**	**Rate based on Target Area Model (molecules of product/catalytic unit/s)**
		**FNR**	**ME**	**FumC**	**AspA**						
**Electrochemistry expt in Figure S8 (20 µL droplet)**	**5**	0.5	0.1	0.1	0.1	16	NA	0.15	0.0094	53.3	90
**Electrochemistry expt in Figure 3 (10 µL droplet)**	**6**	0.1	0.5	0.1	0.1	8	NA	0.21	0.026	26.7	126

Supplementary Table 1 Notes

* Cascade 5FNR: 1ME: 1FumC: 1AspA for all solution assays and electrochemistry experiment 5 (corresponding to Figure S8); overall molecular mass of cascade enzymes = 340,000 Da. In electrochemistry experiment 6 (corresponding to Figure 3) the cascade ratio used was 1FNR:5ME:1FumC:1AspA; total molecular mass of cascade enzymes = 532,000 Da. Molecular masses based on monomers of each enzyme.

* Mediated experiments in solution: 50 mM benzyl viologen, 20 μM $NADP^+$, 20 mM aspartic acid

* Rate given as moles product per total number of moles of enzyme.

* Rates in brackets were obtained as a linear fit to the steepest part of the traces in Figure S13 and as such have not yet reached steady state.

* The volume of a porous electrode of 1 cm x 1 cm x 3 μm is 0.3 μL; for the hypothetical [cascade] in an electrode of these dimensions, the calculation is based on the total volume occupied by the ITO layer: this therefore is an overestimation of the true volume of the pores and as such the predicted concentrations would be even higher.

Supplementary Table 2. List of primers used in the study

Primer	Sequence (5′-3′)
AspA-Forward	GGTGATGATGATGACAAGATGTCAAACAACATTCGTATCGAAGAAGAT
AspA-Reverse	GGAGATGGGAAGTCATTATTACTGTTCGCTTTCATCAGTATAGCGTTT
